# Time management in a co-housed social rodent species (*Arvicanthis niloticus*)

**DOI:** 10.1038/s41598-018-19365-3

**Published:** 2018-01-19

**Authors:** Alexandra Castillo-Ruiz, Premananda Indic, William J. Schwartz

**Affiliations:** 1 Department of Neurology, University of Massachusetts Medical School, Worcester, MA 01655 USA; 2 Present Address: Neuroscience Institute, Georgia State University, Atlanta, GA 30303 USA; 3 Present Address: Department of Electrical Engineering, College of Engineering, University of Texas, Tyler, TX 75799 USA; 4 Present Address: Department of Neurology, Dell Medical School, University of Texas, Austin, TX 78701 USA

## Abstract

Sociality has beneficial effects on fitness, and timing the activities of animals may be critical. Social cues could influence daily rhythmic activities via direct effects on the circadian clock or on processes that bypass it (masking), but these possibilities remain incompletely addressed. We investigated the effects of social cues on the circadian body temperature (Tb) rhythms in pairs of co-housed and isolated grass rats, *Arvicanthis niloticus* (a social species), in constant darkness (DD). Cohabitation did not induce synchronization of circadian Tb rhythms. However, socio-sexual history did affect circadian properties: accelerating the clock in sexually experienced males and females in DD and advancing rhythm phase in the females in a light-dark cycle. To address whether synchronization occurs at an ultradian scale, we analyzed Tb and activity rhythms in pairs of co-housed sisters or couples in DD. Regardless of pair type, co-housing doubled the percentage of time individuals were simultaneously active without increasing individual activity levels, suggesting that activity bouts were synchronized by redistribution over 24 h. Together, our laboratory findings show that social cues affect individual “time allocation” budgets via mechanisms at multiple levels of biological organization. We speculate that in natural settings these effects could be adaptive, especially for group-living animals.

## Introduction

Sociality has beneficial effects on inclusive fitness (e.g., cooperation for resource acquisition or predator defense)^1^, and thus it would follow that natural selection could have favored the evolution of mechanisms that allow individuals within a group to adjust their activity patterns to one another. Theoretically, there are several, mutually non-exclusive, candidate mechanisms for achieving such adaptation. On a daily time scale, social cues might act by synchronizing the endogenous circadian clocks of group members to a common phase or to distinct but stable phase relationships (a mechanism known as entrainment), or by modifying individuals’ rhythm phases and waveforms in ways that may not involve the central circadian clock itself (a mechanism referred to as masking)^2^. Also at shorter (ultradian) time scales, animals might somehow match their activities with others in the group (a masking process referred to as allelomimetism)^3^. Laboratory investigations of the mechanisms by which social interactions might contribute to temporal organization in mammals have been hampered by conceptual and technical difficulties related to animal models (e.g., use of solitary species), assays (e.g., reliance on group activity), and housing conditions (e.g., animals not housed in direct contact), and therefore many effects have appeared small or inconsistent (reviewed in^4^). There have been hints, however, that co-housing animals in direct contact with one another for an extended length of time^5,6^ and controlling for the degree of familiarity of the cohabitants^7^ are important factors.

Here we report our systematic analysis of daily time management in a social rodent species, the Nile grass rat (*Arvicanthis niloticus*), in the laboratory. We view the grass rat, native to Sub-Saharan Africa, as an ideal model to study socially-mediated temporal organization. This species is highly social^8,9^, and breeding pairs show affiliative behaviours (e.g., sitting together, grooming, and caring for pups). Grass rats express a diurnal activity pattern, but individuals can exhibit plasticity of chronotype (i.e., when provided free access to a running wheel, a subset of animals, becomes night-active while the rest remain day-active)^10^. This is a desirable characteristic that can be used as a tool to explore behavioural and physiological plasticity of circadian rhythms. Further, in comparison to other rodents commonly used in circadian studies, grass rats in captivity are more likely to resemble their wild counterparts as there have been continuous efforts to prevent inbreeding; this makes our model more ecologically relevant as the physiology and behaviour of wild animals and their inbred models may not be congruent (e.g.^11^). Much is already known about grass rat circadian rhythmicity, from expression of clock genes in the suprachiasmatic nucleus, to patterns of their locomotor activity rhythms^10,12,13^. To analyze their rhythmicity at circadian and ultradian time scales, we used implantable temperature data loggers (ibuttons) to measure rhythms of body temperature (Tb), as well as passive infrared detectors and video-recordings to determine activity patterns, of pairs of grass rat siblings and heterosexual couples before, during, and after cohabitation in constant darkness and in a light dark cycle. The use of different lighting conditions allowed us to assess effects of social cues on circadian rhythm parameters (period, entrainment phase) as well as on masking.

## Results

### Cohabitation induces changes in circadian period without eliciting synchronization

**Figure 1:**
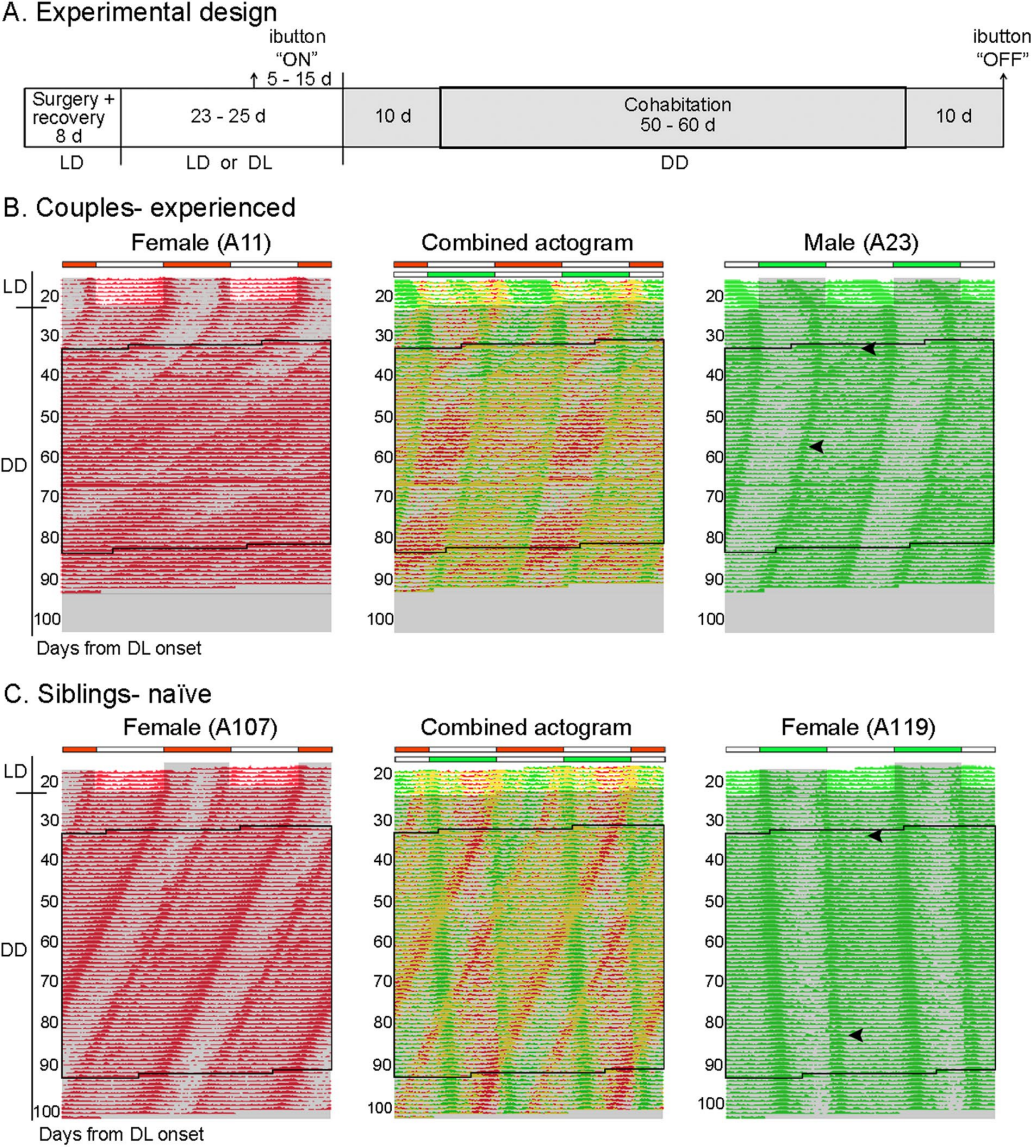
Effects of cohabitation on circadian rhythmicity. (**A**) Timeline of experimental procedures. (**B**,**C**) Representative double-plotted body temperature (Tb) actograms from a sexually experienced couple (**B**) and a sexually naïve female-siblings pair (**C**) showing lack of circadian synchronization. Tb rhythms are plotted as individual (left and right) and combined (middle) actograms. Black-lined box represents the days of cohabitation. Arrowheads on male (A23) and female (A119) actograms indicate the length of time when the rhythm of the other cohabitant is seen on their actograms; this effect was appreciated only during their active phase (i.e., phase-dependent masking). Gray shading indicates darkness.

Female-female and male-male sexually naïve sibling pairs (n = 12 and 10 pairs, respectively; housed together up to the beginning of the experiment) and female-male sexually experienced couples (n = 12 couples; prior to the experiment these animals had formed a breeding pair for ~4 months) underwent the protocol shown in Fig. 1A: after the animals were initially separated and implanted with ibuttons, one member of each dyad was exposed to a reversed light-dark (LD) cycle (DL, phase-shifted) before all animals were released into constant dim red light (DD), then co-housed in DD for about 2 months, and finally separated. Using wavelet analysis of the raw Tb data, we found no evidence for circadian synchronization in any pairing, including some in which all phase relationships between the cohabitants’ rhythms were expressed (i.e., during cohabitation, the Tb rhythm of one animal completely crossed the Tb rhythm of the other; Fig. 1B,C). For the experienced couples, this result was obtained whether the female (n = 9) or the male (n = 12) was phase-shifted before cohabitation.

We were able to rule out the possibility that cohabitation might have led to a type of synchrony in which the locomotor rhythms of the cohabitants actually did synchronize while their Tb rhythms did not. Even though passive infrared detectors on the top of the cages recorded the general locomotor activities of both cohabitants, it was clear that Tb and rest-activity rhythms in individual animals remained congruent, particularly upon separation, when a single rhythm emerged with the characteristics predicted by the appropriate Tb rhythm (Supplementary Figure S1). In a small subset of couples (n = 3) we also tested the possibility that co-housing animals 12 h out of phase might have prohibited their synchronization; but even when these animals were co-housed in phase after being entrained to the same LD cycle from birth, their rhythms drifted apart with different free-running periods (Supplementary Figure S2).

**Figure 2:**
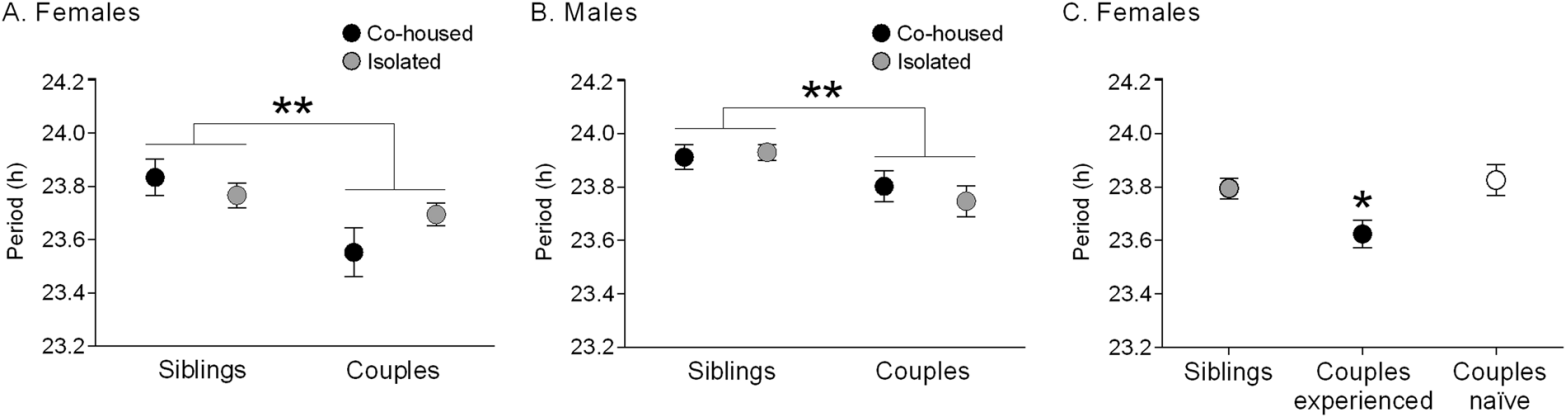
(**A**,**B**) Socio-sexual experience induces a shortening of circadian Tb period in female (**A**) and male (**B**) grass rats regardless of housing condition. (**C**) The effects of socio-sexual history on circadian Tb period in females are not dependent on reproduction. Note that only non-shifted animals (i.e., one member of each pair) were used for calculations of Tb period. Mean ± SEM. *P < 0.05, **P < 0.01.

While our protocol revealed no evidence for circadian synchronization, it did show an effect on circadian period. Grass rats exhibit free-running circadian periods slightly shorter than 24 h in DD^14,15^, but some of our animals were showing notably shorter periods (e.g., the sexually experienced female in Fig. 1B). We therefore compared co-housed females and males with control cohorts of sexually naïve (n = 17 female, 20 male) and sexually experienced (n = 12 female, 13 male) animals kept in isolation for the entire duration of the experiment. The animals that had been phase-shifted before co-housing (i.e., one member of each pair, Fig. 1A) were not included in this analysis due to aftereffects of the phase shift on period (as seen in the >24 h period of female A119 (Fig. 1C)). Likewise, we did not use period before cohabitation in our analysis because of the potential aftereffects of the immediately preceding LD cycle. Two-way ANOVAs were computed separately for females and males, with housing condition (co-housed and isolated) and socio-sexual history (sexually experienced couples and sexually naïve sibling pairs) as independent variables and period after cohabitation as the dependent variable. For both females and males we found a significant effect of socio-sexual history (F(1,49) = 7.89, *P* = 0.007 and F(1,46) = 9.43, *P* = 0.004, respectively), with experienced couples expressing a shorter period than sibling pairs (Fig. 2A,B). In contrast, neither the main effect of housing nor the interaction was significant for females (F(1,49) = 0.35, *P* = 0.56 and F(1,49) = 2.79, *P* = 0.10, respectively) or males (F(1,46) = 0.16, *P* = 0.69 and F(1,46) = 0.60, *P* = 0.44, respectively). Because our sexually experienced animals were generally older than our sexually naïve group, we tested for an effect of age in the isolated sexually naïve control group that was composed of young and older grass rats that matched the age of our experimental animals (2–4 and 6–8 months of age, respectively). We did not identify an effect of age on period (t(33) = 1.19, *P* = 0.24).

Because during cohabitation our couples produced 1–3 litters, and the shortening of period was seen in isolated as well as co-housed sexually experienced animals, this implies that mating, pregnancy, parturition, and lactation during cohabitation also have no effect. To test this, we studied a group of sexually naïve females and males (n = 26) that were first paired at the time of co-housing. Over the cohabitation interval, these couples reproduced successfully with 92% of them producing 1–3 litters, compared to 75% of the experienced couples. In addition, both naïve (62%) and experienced (42%) females showed overt signs of induced estrus ~3 days after males were introduced in the cage as reflected in the scalloping of Tb period every 4–6 days (Supplementary Figure S3). We compared females from these sexually naïve couples to our females from experienced couples and sibling pairs; males were not included in this analysis as they had been phase-shifted. A one-way ANOVA found significant differences between the groups (F(2,63) = 5.03, *P* = 0.009), and posthoc Tukey tests revealed that females in the naïve groups (couples and siblings) were no different from one another (*P* = 0.91) but both groups were different from the experienced couples (*P* = 0.03 and *P* = 0.02, respectively) (Fig. 2C).

**Figure 3:**
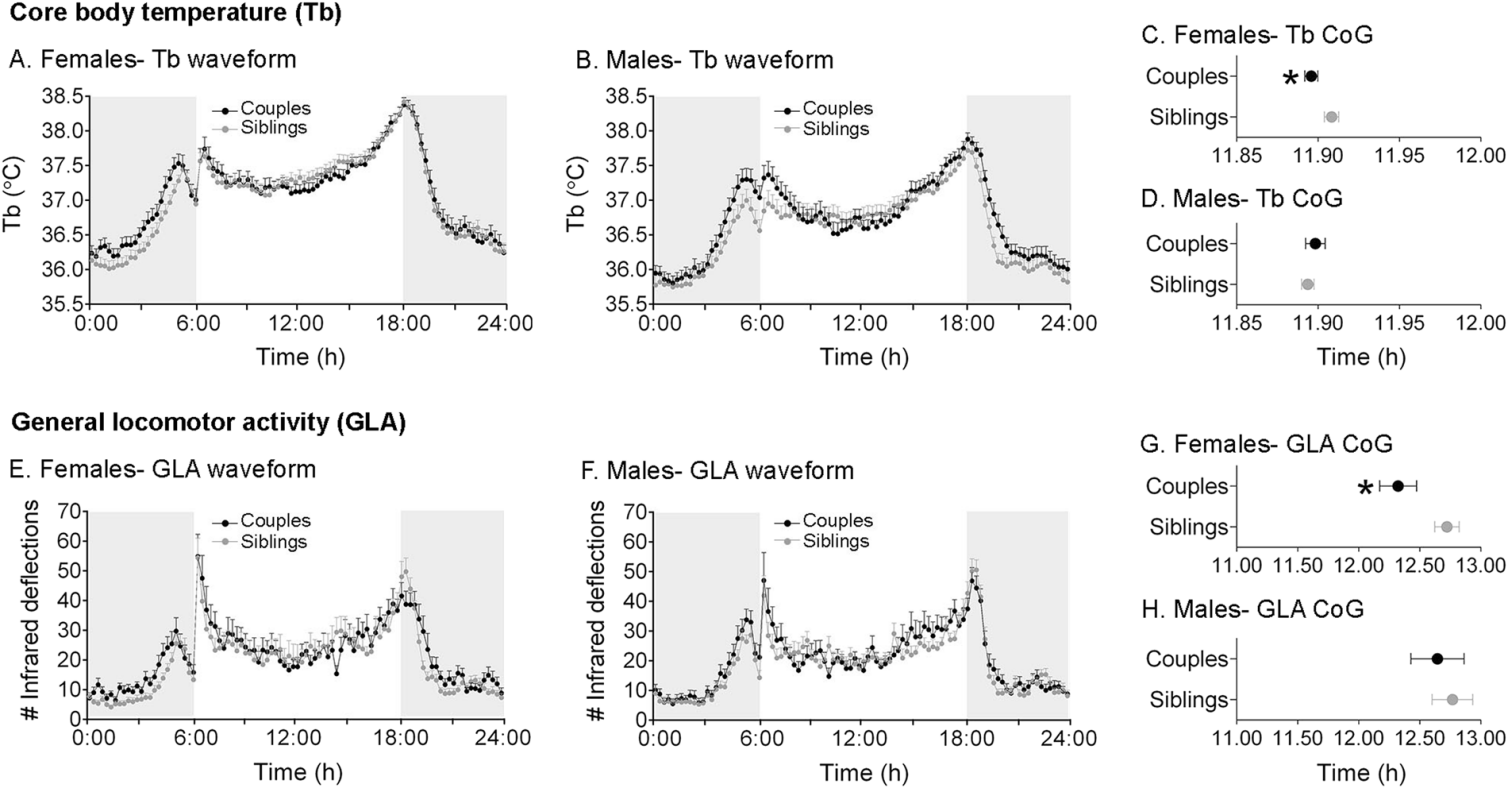
Socio-sexual history is associated with a change in entrainment phase in Tb and general locomotor activity (GLA) rhythms but only in females. Tb (**A**,**B**) and GLA (**E**,**F**) waveforms and associated center of gravity (CoG) (**C**,**D** and **G**,**H** respectively) in isolated female and male grass rats housed as couples (black-filled circles) or siblings (gray-filled circles) prior to the beginning of the experiment. Waveforms and CoG assessments were done with the last 5 days in LD before release into DD (see Fig. 1A). Gray shading indicates darkness. Note that only non-shifted animals (i.e., one member of each pair) were used for calculations of CoG. Mean ± SEM. *P < 0.05.

One possible functional consequence of a shortened circadian period in sexually experienced grass rats would be an advanced (earlier) phase of entrainment to the LD cycle. We tested this by computing the center of gravity (CoG) of the waveforms of Tb and general locomotor activity (Fig. 3) during the 5-day LD interval before release into DD (Fig. 1A). We chose CoG because it is a reliable marker of phase^16,17^. Females from experienced couples expressed a significantly earlier CoG than females from sibling pairs for both Tb (Fig. 3A,C; t(48) = 2.10, *P* = 0.04) and general locomotor activity (3E,G; t(52) = 2.26, *P* = 0.03). Visual inspection of Fig. 3 reveals that the earlier CoG likely reflects an advance in rhythm onset of the sexually experienced females. In contrast to females, males exhibited no difference between the sexually experienced and naïve groups for either Tb (Fig. 3B,D; t(45) = 0.67, *P* = 0.50) or general locomotor activity (Fig. 3F,H; t(50) = 0.46, *P* = 0.65).

### Cohabitation induces synchronization of activity patterns at an ultradian scale

**Figure 4:**
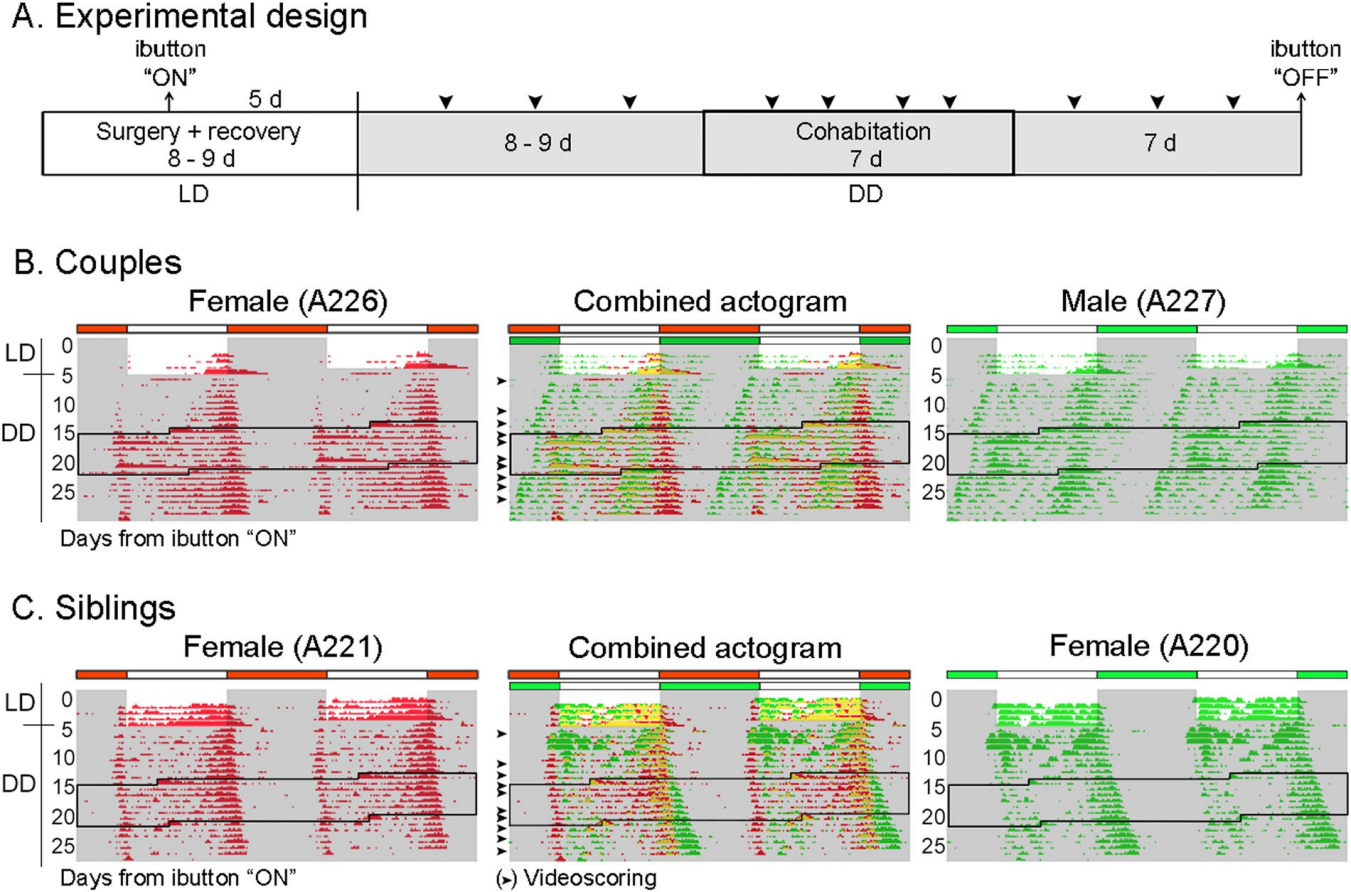
Effects of cohabitation on the temporal organization of activity bouts. (**A**) Timeline of experimental procedures. (**B**,**C**) Representative double-plotted Tb actograms from a couple (**B**) and a female-siblings pair (**C**) confirming no synchronization of circadian rhythms. Tb rhythms are plotted as individual (left and right) and combined (middle) actograms. Arrows represent days of videoscoring. Black-lined box represents the days of cohabitation. Gray shading indicates darkness. Of note, the shift in activity onset of male A227 during cohabitation was not a consistent finding in other pairs. The increase in simultaneous activity seen in (**B**) (at a 3–4 day interval) during cohabitation is likely driven by the female’s estrous cycle (see text).

Some of our cohabitation actograms appeared to show an “imprint” of one animal’s Tb rhythm on the “subjective day” of its cohabitant (e.g., in Fig. 1, the rhythm of female A11 is seen on the actogram of male A23 (Fig. 1B), and female A107 on female A119 (Fig. 1C)). To further investigate such rhythm “masking” and its possible relationship to rest-activity bouts, sexually naïve females (n = 15) and males (n = 5) underwent the protocol shown in Fig. 4A: after the animals were initially separated from their siblings and implanted with ibuttons, they were released into DD, then co-housed as female-male couples or female-female pairs (n = 5, respectively) for 7 days, and finally separated. The resulting Tb actograms, even though of short duration, confirmed a lack of circadian synchronization (Fig. 4B,C). In most cases the animals showed a lengthened circadian period which was likely associated with the higher dim red light intensity to which the animals were exposed for video-scoring. This is consistent with reports in grass rats showing that increases in light intensity lengthen free-running period^14,18^.

**Figure 5:**
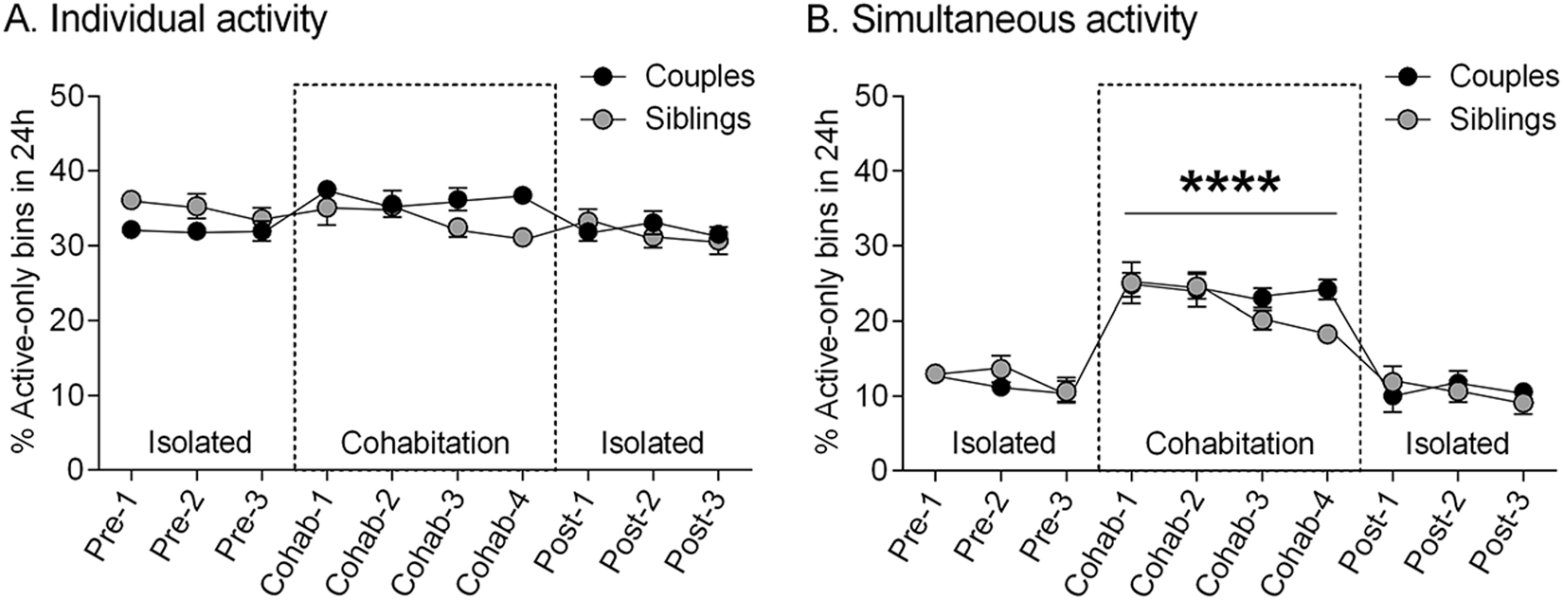
Cohabitation did not induce changes in the percentage of time individuals from couples (black-filled circles) or female-siblings pairs (gray-filled circles) were active per day (**A**) but double the percentage of time the grass rats were simultaneously active per day, regardless of pair type (**B**). Dotted box represents the days of cohabitation. Mean ± SEM. ****P < 0.0001.

Individual and simultaneous locomotor activity was tabulated by videoscoring each animal as either active or inactive at 5 minute intervals for 3, 4, and 3 non-consecutive days before, during, and after cohabitation, respectively (Fig. 4A; note that sexual behavior was not included in the analysis). The “percent of active-only bins” over each 24 h span was calculated as the number of active scores divided by the total number of scores (active + inactive) × 100. Simultaneous activity was calculated as the percent of active-only bins for which both members of a pair were active at the same time; the estimated simultaneous activity before and after cohabitation, when the animals were separated, was calculated by virtually superimposing their individual activity profiles for those 24 h spans. A repeated measures ANOVA was conducted to determine the effect of cohabitation on individual and simultaneous activity patterns with sampling day as the repeated measures variable and group (couples and sibling pairs) as the between measures variable. The analysis for individual activity (Fig. 5A) revealed no significant effect of group (F(1,18) = 0.11, *P* = 0.75); animals in both groups were individually active about 1/3 of the time per 24 h. However, there was a significant effect of sampling day (F(9,162) = 4.29, *P* < 0.0001) and of the interaction F(9,162) = 4.40, *P* < 0.0001); siblings showed a decrease in activity as the experiment progressed, while couples increased their individual activity slightly during cohabitation compared to pre- and post-cohabitation (Sidak’s test), which suggests that socio-sexual cues may also influence individual activity levels. On the other hand, the analysis for simultaneous activity (Fig. 5B) showed a significant effect of sampling day (F(9,72) = 43.28, *P* < 0.0001), and post-hoc analyses revealed an increase in simultaneous activity during cohabitation (Tukey, all *P*s < 0.0001); the percentage of time that animals in both groups were simultaneously active was double what would have been expected pre- and post-cohabitation. The effect of group or the interaction was not significant (F(1,18) = 0.14, *P* = 0.72; F(9,72) = 1.56, *P* = 0.14, respectively).

## Discussion

We analyzed the effects of laboratory cohabitation on circadian and ultradian rhythmicity of grass rats. Our design included direct physical contact between animals co-housed as heterosexual couples or same-sex pairs, without daily interference or ambient lighting cues, for an extended length of time; our intent was to provide conditions as favorable as possible for eliciting social influences on the temporal organization of the cohabitants.

Nevertheless, we found no evidence for synchronization of circadian rhythmicity, as measured by the body temperature rhythm; this was the case even when animals were paired in phase after entrainment to the same LD cycle. At least one other free-running rhythm (locomotor activity) also failed to synchronize between animals, and did not desynchronize from the temperature rhythm within animals, a phenomenon reported under certain lighting conditions^19–21^. Our experience is consistent with previous reports, sp., that mutual circadian synchrony between mammalian pairs in the laboratory is unusual (reviewed in^4,22–24^). A key to achieving such synchrony may be by grouping a larger number of animals together^6,25^.

The fact that our female-male couples, whether sexually experienced or naïve, enjoyed reproductive success–despite free-running with different circadian periods – is reminiscent of classic experiments by Richter^26^. He reported successful mating in blinded rat pairs that exhibited intersecting circadian periods of less than and greater than 24 h, only when their active (subjective night) phases overlapped.

It is known that circadian period can be modulated by social cues^5,27^. Here we also show period modulation, but in an unexpected way: regardless of sex or housing condition, sexually experienced grass rats expressed a significantly shorter free-running period than sexually naïve animals. This period shortening must be a delayed effect of their coupling, because sexually naïve females and males that were paired–and experienced mating, pregnancy, parturition, and lactation during their 2 month cohabitation–did not exhibit such a period change. That a stimulus may have a delayed effect on behaviour is not unknown, e.g., the mating-induced change in male mouse infanticidal behaviour^28^. Although the long-lasting mechanism for the socio-sexual induction of a faster clock remains to be elucidated, one possible candidate is an increased level of arousal which is known to shorten circadian period in other rodents^29^, and we observed a small but statistically significant increase in individual activity bouts exhibited by couples but not by siblings during cohabitation.

Sexually experienced females also exhibited an advanced entrainment phase to the LD cycle, a chronotype consonant with a shorter circadian period in DD (e.g.^30,31^). This feature was not observed in sexually experienced males, suggesting that oscillator properties other than period contribute to entrainment phase^32^. The mechanism(s) underlying the changes in period and phase seen in females remain to be elucidated; however, ovarian estrogens are one plausible candidate as they are known to shorten period and advance the onset of activity in various rodent species^33,34^. Thus perhaps in our sexually experienced female grass rats, mating induces a delayed and protracted increase in background levels of estradiol. But, what would be the functional significance of a change in period and phase? From an evolutionary point of view, phase of entrainment, not period, is a parameter under selection^35^. During the mating season, female grass rats are continuously challenged with pregnancy and simultaneous lactation, which impose high energetic demands in small rodents^36^ and may be associated with switching of the temporal niche^37^. Even though the difference in phase between naïve and experienced female grass rats seems modest, an earlier entrainment phase in the range of minutes can have a critical impact on resource acquisition^38^ and reproductive fitness^39^ in the wild. For example, work in great tits shows that a delay in activity onset of 10 minutes in males increases the risk of being cuckolded^39^. On the other hand, in blue tits, males that begin their dawn chorus 6 minutes earlier on average than the rest have more mating partners and are likely to sire more offspring^40^. Therefore, for the future, it will be interesting to test the possible functional significance of our laboratory findings in natural settings. We speculate that in the field, once a female grass rat affiliates with a male, an advanced phase can confer a fitness advantage. Because in the wild grass rats live in an environment in which temperature fluctuates dramatically throughout the day and because they show hyperthermia during much of gestation and lactation^41^, we propose that the change in chronotype seen in sexually experienced females reflects a mechanism that minimizes energy expenditure through avoidance of non-thermoneutral temperatures and maximizes resource acquisition. In fact, modifications in temporal activity patterns are seen as an adaptation to environmental temperature fluctuations^42^.

In our final experiment, we asked whether grass rat dyads actually do synchronize their activity/rest patterns, but at an ultradian rather than circadian scale. Our finding of a cohabitation-associated increase in the simultaneity of activity bouts suggests that co-housed animals re-distribute their activity to coincide with one another. We are aware of a report of socially synchronized ultradian rhythmicity in a semi-natural field study of voles under snow, in the absence of photic entrainment^43^, and perhaps in the laboratory under an LD cycle^44^. The mechanism(s) are uncertain; however, phase-dependent masking brought about by social cues has been observed in other rodents^45,46^, and there is evidence of phase-dependent effects on behaviour in nature, e.g., groups of meerkats and chacma baboons show high behavioural synchrony in the morning^47,48^. For the future, a fine-grained analysis of the specific activities of cohabiting grass rats may reveal phase-dependent effects.

In sum, our results in the laboratory reveal that the effects of social interactions on temporal organization are complex and exerted at multiple levels of biological organization. Indeed, a recent study of avian biparental incubation rhythms revealed surprising within- and between-species diversity in period length^49^. Of note, despite the presence of daily environmental cues, 24 h rhythms were absent in 78% of the nests (representing 18 of 32 species). Future further integration of laboratory and field chronobiology will surely be key to new insights on the collective synchronization of animals living in groups.

## Methods

### Animals

Grass rats of various ages were obtained from our breeding program at the University of Massachusetts Medical School. Animals were maintained in 12 h: 12 h light-dark (LD; lights on 06:00, off 18:00) cycles with *ad libitum* access to food and water. All animal procedures were in accordance with the National Institutes of Health Guide for the Care and Use of Laboratory Animals and approved by the Institutional Animal Care and Use Committee of the University of Massachusetts Medical School.

### ibutton implantation

ibuttons (DS1922L, Maxim Integrated Products, Inc., San Jose, CA, USA) were programmed to record body temperature every 15 min beginning 17–26 days after implantation (Fig. 1A) or every 5 minutes beginning 3–4 days after implantation (Fig. 4A) for a total of 85 and 28 recording days, respectively. ibuttons were coated in paraffin/elvax wax (Mini Mitter, Sunriver, OR, USA), sterilized, and implanted in the peritoneal cavity under isofluorane anesthesia. Animals received buprenorphine (0.05 mg/kg) and ketoprofen (5 mg/kg) subcutaneously at the time of surgery and meloxicam (0.4 mg/kg) orally 24 h and 48 h after surgery.

### Effects of cohabitation on circadian rhythmicity

Female-female and male-male sexually naïve sibling pairs (44 grass rats, 3 months of age) and female-male sexually experienced couples (24 grass rats; 12 couples; 6–10 months of age, paired for ~4 months before the beginning of the experiment) were separated 1–2 days or 1–53 days, respectively, prior to surgery. The longer times applied to dams that were pregnant at separation; for these females we waited until weaning (21 days) to proceed with surgery. Eight days after surgery, one member of each dyad was phase shifted by 12 h (reversed LD cycle; lights on 18:00, off 06:00) while the other member remained in the previous LD cycle, and 23–25 days later all animals were released into constant dim red light (DD; light intensity was on the order of 300–400 lux for LD or DL, and <3 lux for dim red, at the mid-cage level). After 10 days, pairs and couples were co-housed for 50–60 days, followed by a final 10 days of separation before euthanasia and collection of ibuttons (experimental chronology in Fig. 1A). An additional group consisted of sexually naïve females and males (26 grass rats; 1–5 months of age) that underwent the same protocol as above and were then paired at the time of co-housing. Control cohorts of sexually experienced (12 female, 13 male; 6–10 months of age) and sexually naïve (17 female, 19 male; 2–8 months of age) grass rats were kept in isolation for the entire duration of the experiment. General locomotor activity was monitored via passive infrared detectors (K-940, Visonic, Tel-Aviv, Israel) positioned on the top of the cages. These data were binned every 5 min by using the Vitalview Program (Philips Respironics, Bend, OR, USA).

Temperature actograms were created with the Activeview Program (Philips Respironics) by plotting the values obtained after subtracting the mean and two standard deviations from each individual data point $({X}_{i}-(\bar{X}-2(S))$, where *X*_*i*_ = individual data point, $\bar{X}$ = mean for entire data set and *S* = standard deviation for entire data set). The period of the free-running circadian temperature rhythm in DD was determined by drawing an eye-fitted regression line over onsets and offsets spanning 9 days after cohabitation using the tau cursor function in the Activeview Program. Phase of entrainment to the LD cycle for the 5 days prior to DD preceding cohabitation was assessed by computing the center of gravity (CoG) for the body temperature and general locomotor activity waveforms, as described previously^50^. Circadian period and phase during cohabitation was assessed by transforming the raw temperature data using the Mexican Hat continuous wavelet transform to determine the cycle-to-cycle phases of the circadian offset and peak and their difference with respect to a reference phase, as described previously^51^. This analysis allowed for the prediction of the expected trajectories of the rhythms over the duration of the experiment (as extrapolated from the 10 days in DD before co-housing).

### Effects of cohabitation on the temporal organization of activity bouts

Sexually naïve female and male grass rats (15 females, 5 males; 2–4 months of age) were separated from their siblings for 1–2 days before surgery, fitted with ibuttons, and then released into DD 8–9 days after surgery. After 8–9 days of free run, female-male couples (5 pairs) and female-female pairs (5 pairs) were co-housed for 7 days, followed by a final 7–8 days of separation before euthanasia and ibutton collection. Activity patterns were analyzed by scoring active-only behaviours (note: reproductive behaviour was not scored) every 5 min for 3, 4, and 3 non-consecutive days before, during, and after cohabitation, respectively (experimental chronology in Fig. 4A). Animals were videotaped using videocameras (BO9880DN, Sony, Tokyo, Japan) fitted with varifocal lenses (TG3Z2910FCS, Computar, CBC Co., Ltd., Tokyo, Japan) connected to a surveillance system (DR4HD/500, Ganz, CBC Co., Ltd., Tokyo, Japan), and additional light sources (red lamps) were used to aid with behavior scoring (light intensity was on the order of ~5 lux for dim red, at the mid-cage level). General locomotor activity was monitored as described above. Individual activity was calculated as percent of active-only bins per 24 h interval, and simultaneous activity was calculated by assessing the percent of active-only bins for which both members of a pair were active at the same time per 24 h interval. The estimated percent of simultaneous activity before and after cohabitation, when the animals were separated, was calculated by virtually superimposing their individual activity profiles for that 24 h interval.

### Data analyses

One-way and two-way ANOVAs as well as t-tests (two-tailed) and posthoc tests (Tukey and Sidak) were computed using GraphPad Prism version 6 for Windows (GraphPad Software Inc., La Jolla, CA, USA). Wavelet analysis was performed using MatLab (The MathWorks, Inc., Natick, MA, USA). Significance was assumed if *P* < 0.05.

## Electronic supplementary material

Electronic supplementary material
