# Supplementary material for: Time management in a co-housed social rodent species (Arvicanthis niloticus)
Source: Sci Rep. 2018 Jan 19;8:1202. doi: 10.1038/s41598-018-19365-3 (PMC5775316; doi:10.1038/s41598-018-19365-3)

Time management in a co-housed social rodent species (*Arvicanthis niloticus*)

Alexandra Castillo-Ruiz, Premananda Indic, William J. Schwartz

Supplemental Figure Legends

Supplementary Figure S1. Tb and GLA double-plotted actograms of a representative female grass rat from the female-siblings pair shown in Figure 1. The Tb (left) and GLA (right) rhythms did not show signs of internal desynchronization as both rhythms completely mirrored each other throughout the experiment. The second rhythm that appears on the GLA actogram during cohabitation corresponds to the other member of the dyad. Black-lined box represents the period of cohabitation. Gray shading indicates darkness.

Supplementary Figure S2. Tb double-plotted actograms of a representative couple entrained to the same LD cycle prior to release into DD. Tb rhythms are plotted as individual (left and right) and combined actograms (middle). Black-lined box represents the period of cohabitation. Gray shading indicates darkness.

Supplementary Figure S3. Tb double-plotted actogram of a representative female grass rat showing scalloping (asterisks) of the Tb rhythm 3 days after the male was introduced into her cage (arrow). This phenomenon was repeated every 5 days until the female became pregnant (at last asterisk).

Supplementary Figure S1.

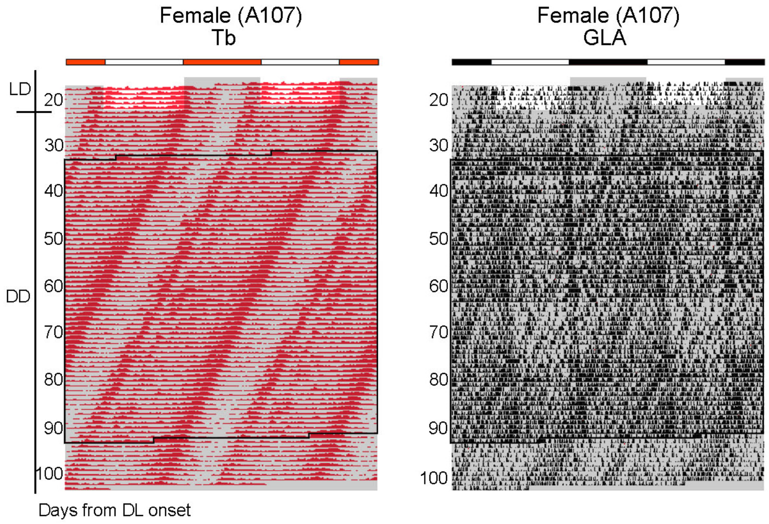

Supplementary Figure S2.

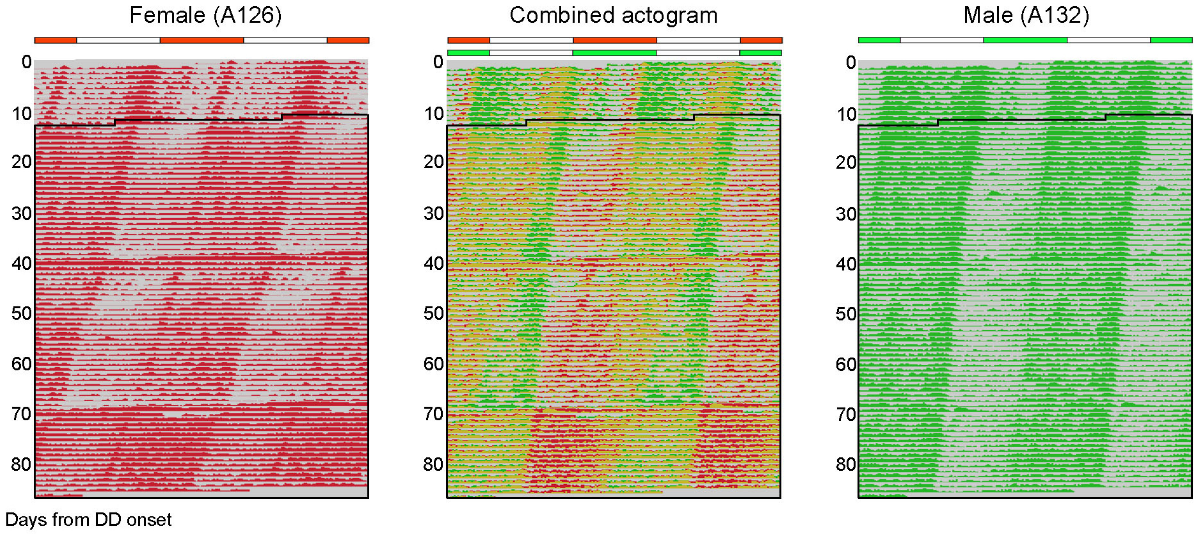

Supplementary Figure S3.

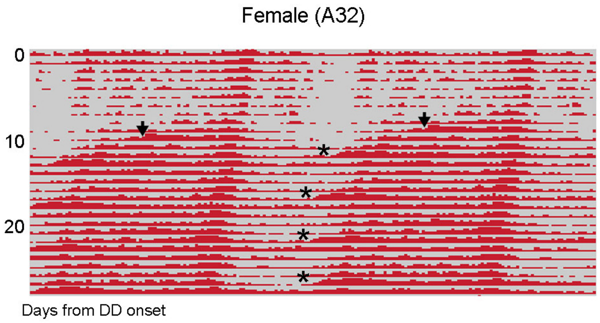

Supplement: Supplementary file 1 — Electronic supplementary material [file 41598_2018_19365_MOESM1_ESM.pdf]
